# TRANSFoRm Data quality tool

**DOI:** 10.1186/2043-9113-5-S1-S15

**Published:** 2015-05-22

**Authors:** Robert A Verheij

**Affiliations:** 1 NIVEL, Netherlands Institute for Health Services Research, 3513 CR Utrecht, the Netherlands

## Characterisation

Data quality, primary care databases.

## Tool description

As computerisation of primary care facilities is rapidly increasing, a wealth of information is created in routinely recorded electronic health records (EHR) that can be used and is already used for research purposes. However, we need to be able to assess whether the data these primary care databases contain is ‘fit for purpose’.

In the TRANSFoRm project an axiomatic approach was developed that started with defining purpose and population, followed by the definition of a set of metrics for data quality described in terms of completeness, accuracy, correctness and consistency.

**Figure 1:**
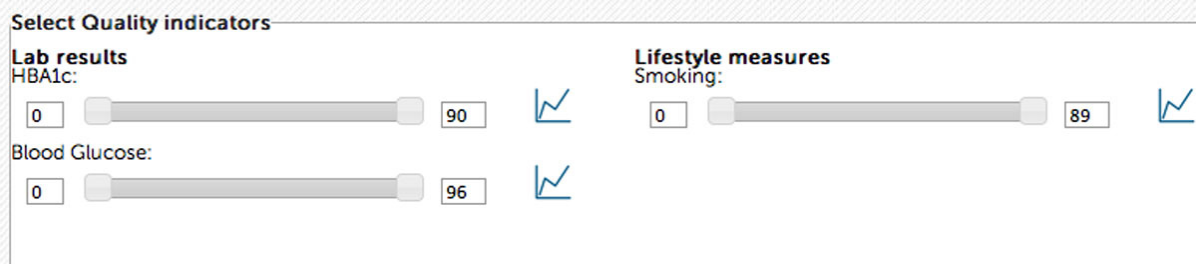
User interface of the Quality Tool indicating results for three data types. The moving of the slide bars results in the selection of primary care sites where all criteria are met, allowing a researcher to weigh data quality against a desired number of practices. The tool is an example of how different metrics can be used to select data that is fit for purpose.

Based on this approach a web-based prototype of a data quality tool [1] was developed and evaluated in two TRANSFoRm clinical use cases (GORD and Diabetes), allowing researchers to select primary care sites with data that is ´fit for purpose´. For example, a researcher might only be interested in practices with more or less complete recordings of HbA1c, blood glucose and smoking status (Figure 1).

## Status of development

Prototype.

## Users

Members of the TRANSFoRm project; the tool will be built into the TRANSFoRm query workbench.

## Link

http://www.transformproject.eu/
